# Selection, intake, and plate waste patterns of leftover food items among U.S. consumers: A pilot study

**DOI:** 10.1371/journal.pone.0238050

**Published:** 2020-09-09

**Authors:** Brian E. Roe, Danyi Qi, John W. Apolzan, Corby K. Martin

**Affiliations:** 1 Department of Agricultural, Environmental and Development Economics, Ohio State University, Columbus, OH, United States of America; 2 Department of Agricultural Economics and Agribusiness, Louisiana State University, Baton Rouge, LA, United States of America; 3 Pennington Biomedical Research Center, Louisiana State University System, Baton Rouge, LA United States of America; Universidad de Los Andes, COLOMBIA

## Abstract

Many campaigns promote the preservation and consumption of leftover food items as a critical household strategy to accomplish national consumer food waste reduction goals. We fill a gap in knowledge about the consumption and creation of leftovers in the United States by analyzing data from a pilot study in which 18 subjects tracked food selection, intake, and plate waste across all eating occasions for about one week. Subjects noted which items selected for consumption were leftovers, i.e., previously prepared but uneaten items that were stored for future consumption, and which unfinished items were saved to become leftovers. We found that 12% of items selected for consumption were leftovers while 24% of selected items that were not fully consumed were kept to become a leftover. Leftovers were most frequently vegetables, cheeses, and meats, and most frequently selected on Mondays and for lunch. Regression analyses isolate significant dining patterns with respect to leftovers, including evidence that leftovers were less likely to be fully consumed than non-leftover items, and that larger meals led to more uneaten food. This suggests that strategies to reduce meal size may be most effective in reducing food waste by limiting the creation of leftovers in the first place. Strategies to make leftovers more attractive and appealing may also reduce food waste.

## Introduction

Reducing the amount of food that is wasted has become a policy goal of numerous national governments [1,2] and of the United Nations through its Sustainable Development Goals [3]. Food waste reduction goals are seen as a way to improve food security through the consumption of nutrients and calories that would otherwise be discarded; enhance sustainability by reducing the negative environmental and resource impacts of foods that are produced, transported, stored then landfilled; and strengthen financial outcomes by more efficiently converting dollars spent into foods consumed.

The campaigns developed to support achievement of these goals, e.g., [4], feature modules focused on consumers and, without exception, these modules urge consumers to keep foods and food ingredients that are unused after the preparation and completion of meals (hereafter, leftovers saved) and select these items for subsequent meals (hereafter, leftovers selected). While leftovers are a ubiquitous focus of consumer food waste reduction campaigns, most efforts also encourage other consumer actions, such as careful meal planning and appropriate selection of portion and meal sizes such that leftovers are minimized. However, the extant literature provides little guidance on which consumer-focused strategies would yield the greatest food waste reduction.

Few examples of research focused on leftovers at the household level have been published. Several studies consider leftovers through a sociological lens and explore these themes through ethnographic methodologies. In general, this literature emphasizes leftovers in the context of household food provisioning routines and the social relations that shape meal and food conventions within a household [5–9]. Themes include the consumption of leftovers as a sacrifice for the family [5], leftover avoidance due to perceived demand among household members for tried and tested recipes rather than new recipes required to ‘use up’ leftovers [6], leftover storage in the refrigerator as a guilt deferral tactic [7], and poor storage tactics leading to leftover decay and waste in home refrigerators [8,9].

Other veins of literature quantify consumer views and perceptions of leftovers. Aschemann et al. [10] note that most consumers view eating leftovers as a trade-off or compromise. Parizeau et al. [11] ask consumer respondents how frequently they use leftovers and find a negative association between leftover use and household food waste. This corresponds with the finding by Stancu et al. [12] who document that respondents who report frequent use of leftovers also report lower levels of wasted food. Van der Werf et al. [13] elicit agreement with several statements concerning leftovers (e.g., consuming leftovers is safe, it is difficult to use leftovers to create new meals) among survey respondents and use these responses to develop constructs related to their model of the theory of planned behavior.

Guiding the prioritization of food waste reduction strategies, e.g., understanding whether leftover attendance or meal planning is more efficacious, would require data that links household food consumption and waste behavior to key consumer strategies such as saving and selecting leftovers and meal size management. However, to the best of our knowledge, the only data quantifying household leftover behavior is taken from retrospective self-reports provided by online consumer panelists in the United Kingdom for midday and evening meals [14], where the accuracy of self-reported food intake data have been questioned [15–18].

To begin to fill this void, we summarize detailed food selection, intake, and plate waste data collected by 18 consumers over the course of approximately a week during a pilot study conducted to refine a smartphone app designed to collect such data. The subjects denoted whether each of the 1,177 individual items that were selected across all the meals and snacks they ate were leftovers and whether items left unfinished at the end of the meal were being saved as a leftover. The data gathered include detailed information on the amount and type of foods, which permits the first insights into meal behaviors and patterns concerning leftovers and permits analysis at a granular level. Specifically, we describe the frequency of leftover selection and leftover creation, the types and situations when leftovers are selected and created, and isolate via regression analysis whether the selection of leftover items as part of a meal is associated with caloric intake and plate waste amounts for both the leftover item and for non-leftover items selected as part of the meal.

## Materials and methods

### Sampling

The study sample includes 18 adults (3 male, 15 female) age 33 to 60 years (m = 50.8, sd = 8.2) recruited from the region including Baton Rouge, Louisiana. Participants needed to be able to consent, be 18–72 years old, have a body mass index (BMI) between 18.5–50 kg/m^2^, have an iPhone, and be willing to download an app and use it for the duration of the study. Participants could not be pregnant or planning to become pregnant while enrolled in the study. Participants’ BMI was calculated prior to initiating the Remote Food Photography Method© (RFPM, detailed below) using height and weight captured in a clinical research unit on a calibrated stadiometer and scale. The prevalence of female respondents may be driven by the combination of the focus of the study (tracking food selection and waste) and the fact that U.S. women still spend more than twice the time in household meal preparation as men, creating a lower perceived participation burden [19]. The study was approved by the Pennington Biomedical Research Center IRB. Participants received compensation for participant in the research.

### Measurement

Participants in their free-living conditions recorded data during nearly all eating occasions for about one week. Eating occasions covered all times of day, weekdays and weekends, and food away from home as well as food at home. Data analysis followed the RFPM© (see [20,21] for a full description and discussion of its validation). Data were collected using the SmartIntake® app. Briefly, using smartphones containing the app, participants capture photos of the food they selected before each eating episode and their plate waste after each eating episode including any occurrences of multiple servings (e.g., second helpings) and any consumption of caloric beverages. Participants also collected data about any food scraps generated during home preparation of meals, which will be analyzed and reported elsewhere. As data was acquired, it was monitored in near real-time. If a respondent failed to send data, the participant (on their own or at the request of the researchers) submitted self-reported data recorded either on a paper diary or entered electronically into the SmartIntake® app interface that was then sent to the researchers with the mode of recording chosen according to the respondent’s preference. Previous studies using the SmartIntake app reveal no significant change in participant weight or energy intake (as assessed by doubly labeled water method) during the study period, suggesting that the app is not inducing changes to base eating patterns [21]. Our participants record an average of 3.3 eating occasions per day during the study period (meals or snacks where food is ingested, but excluding occasions that only involve drinks), which is greater than that documented by the American Time Use Study (2.7) but less than the National Health and Nutrition Examination Survey (4.4 per day, excluding drinks) [22], which is generally considered more comprehensive.

If a food’s identity on the image was unclear, the participant identified the food through an interface in the smartphone app to help us link the food to a nutrient database; specifically, the United States Department of Agriculture’s (USDA) Standard Reference 28 [23] and the Food and Nutrient Database for Dietary Studies, FNDDS, 6.0 [24] were in use when these data were collected. The photos are automatically received by our server for analysis, where the research team uses a computer-assisted approach to identify a match for each food in a nutrient database and estimates portion size based on established and validated procedures [20, 21, 25, 26]. The analyst enters the portion size for food selection and plate waste, and the computer system calculates the energy and nutrient composition for food selection, plate waste, and food intake, where food intake is calculated as selection minus waste.

The foods were then assigned to one of ten food groups for data analysis based on product content and preparation characteristics: (1) baked products (e.g., bread and pastries), (2) breakfast cereals, grains and pasta, (3) dairy and egg products, (4) fast food and restaurant food, (5) fruits and fruit juices, (6) nuts, seeds, and legumes, (7) fats, oils, soups, sauces, gravies, spices and herbs, (8) meat and meat products, (9) sweets, and (10) vegetable and vegetable products.

### Analysis

Results are analyzed in Stata (version 14.2). Food item variables include the number of servings selected, grams selected, caloric density (calories per serving), and food group. Items that were beverages were excluded from the analysis. The definition of servings for each type of food is based upon the most appropriate portion size image; in some cases this leads to a high serving count for a small amount of food (e.g., one baby carrot is counted as one serving). Thus servings are not necessarily manufacturer standard servings nor USDA standard servings, but are likely the standard that allows for the best portion size estimation (i.e. a smaller portion will likely have a smaller serving whereas a larger portion will likely have a larger serving).

Statistics and cross-tabulations at the level of individual food items are presented in Tables 1 and 2 and at the level of the eating occasion (meals and snacks) in Table 3. Differences in proportions across groups are determined with a chi-square test. Table 4 contains multivariate regression analyses explaining the waste rate, the probability of any waste (a linear probability model, see [27]), calories selected and calories consumed. The application of regression analyses permit isolation of the independent role of explanatory variables (e.g., selection of leftovers, meal size) on these dependent variables, which provides additional insights compared to the significance of tests of cross tabulations that may capture the association of other variables that are correlated with the factors being tested. The regression analyses are performed via ordinary least squares and feature standard errors clustered at the subject level. Unconsumed items that were designated as future leftovers were not included. Individual fixed effects are included to control for differences across participants that are time invariant. Study day fixed effects are included to control for differences that are common to each successive day of participation. The minimum relative humidity on the day of the meal was included to control for the potential influence of daily weather patterns on food selection [28]. Statistical significance is denoted at the 5% and 1% levels, while results with *p*-values between 5% and 10% are denoted as marginally significant.

**Table 1:** Food items by leftover and consumption status.

		The item was fully consumed during the meal	Item was not fully consumed during the meal and was…		Row Total
			…kept as a leftover	…not kept as a leftover	
When the item was selected it was:	A Leftover	129	3	11	143
		(10.96%)	(0.25%)	(0.93%)	(12.15%)
		[13.16%]	[6.25%]	[7.38%]	[12.15%]
		{90.21%}	{2.10%}	{7.69%}	{100%}
	Not a Leftover	851	45	138	1034
		(72.30%)	(3.82%)	(11.72%)	(87.85%)
		[86.84%]	[93.75%]	[92.62%]	[87.85%]
		{82.30%}	{4.35%}	{13.35%}	{100%}
Column Total		981	48	149	1177
		(83.26%)	(4.08%)	(12.66%)	
		[100%]	[100%]	[100%]	
		{83.26%}	{4.08%}	{12.66%}	

Numbers in each cell represent number of items, (percent of total items), [percent of items in the column], and {percent of items in the row}. Pearson χ^2^(2) = 5.68 (*p* = 0.058).

**Table 2:** Leftovers selected and saved as a percent of items, by food, meal and participant characteristics.

Categories	Leftovers Selected	N	Leftovers Saved Among Items not Fully Consumed	N
Total: % of Items	12.15%	1,177	24.37%	197
[% of calories]	[9.71%]		[28.06%]	
By Food Type	(*p = 0*.001***)		(*p* = 0.567)	
Baked Products	10.09%	109	21.05%	19
Breakfast Cereals, Grains, & Pasta	16.25%	80	35.71%	14
Dairy & Egg Products	8.23%	158	23.53%	17
Fast Foods & Restaurant Foods	7.06%	85	10.34%	29
Fruits & Fruit Juices	6.90%	87	23.08%	13
Nut, Seeds, & Legumes	5.17%	58	0.00%	3
Fats, Oils, Soups, Sauces, Gravies, Spices, & Herbs	11.50%	113	22.58%	31
Meats & Meat Products	16.03%	156	33.33%	24
Sweets	5.26%	76	20.00%	10
Vegetables & Vegetable Products	19.22%	255	32.43%	37
By Eating Occasion	(*p*<0.000***)		(*p* = 0.563)	
Breakfast	4.78%	251	12.90%	31
Morning Snack	0.00%	25	33.33%	3
Lunch	19.11%	429	26.14%	88
Afternoon Snack	9.26%	54	16.67%	6
Dinner	10.88%	377	28.36%	67
Evening Snack	7.32%	41	0.00%	2
By Day of the Week	(*p*<0.000***)		(*p* = 0.016**)	
Monday	23.75%	160	12.20%	41
Tuesday	8.33%	168	40.63%	32
Wednesday	13.94%	165	33.33%	24
Thursday	10.11%	188	34.62%	26
Friday	12.74%	157	33.33%	18
Saturday	10.80%	176	13.79%	29
Sunday	6.15%	163	11.11%	27
By Gender	(*p*<0.000***)		(*p* = 0.420)	
Female	10.47%	993	24.62%	195
Male	21.20%	184	0.00%	2
By Age	(*p*<0.000***)		(*p =* 0.001***)	
< 40	2.65%	113	10.00%	40
40–49	7.17%	321	34.62%	26
50–59	13.10%	580	20.39%	103
60 and older	25.15%	163	50.00%	28
By an Item’s Waste Rate	(*p =* 0.149)		(*p* = 0.104)	
None	13.16%	980	20.27%	148
<25%	8.47%	59	40.00%	35
25% - 49.9%	7.14%	56	27.27%	11
50% - 74.9%	3.36%	55	33.33%	3
75% or more	11.11%	27	20.27%	148
By Calories Selected for Occasion	(*p = 0*.000***)		(*p* = 0.032**)	
<250	4.63%	216	21.74%	23
250–499	10.00%	320	16.28%	43
500–749	21.01%	338	12.82%	39
750 or more	9.90%	303	33.70%	92

Example interpretations are as follows (see line for baked products): 10.09% of the 109 baked product items selected by respondents for meals were identified as leftovers while 21.05% of the 19 baked product items that were selected but not fully consumed were being saved to become a leftover. *p*-values are from chi-square tests of association within each category.

**Table 3:** Summary statistics by eating occasion (meals and snacks).

VARIABLES	Meals (N = 307)	Snacks (N = 41)
% of occasions featuring at least one leftover	11.73%	2.44%
Among meals with at least one leftover selected, % of meal’s calories from leftovers	79.19%	--
	(0.285)	
	(N = 36)	
% of occasions generating a leftover	5.86%	2.440%
Among occasions with unconsumed items, % generating a leftover	19.77%	25%
	(N = 86)	(N = 4)
Average # of items selected per occasion (SD)	3.45	1.61
	(2.14)	(1.07)
Average kcal selected per occasion (SD)	468.93	251.68
	(321.57)	(150.22)

**Table 4:** Regression model results among items not saved for later.

	(A)	(B)	(C)	(D)
VARIABLES	OLS of Food Waste Rate for this item	LPM = 1 if any remains uneaten	OLS Kcals of the item selected	OLS Kcals consumed of the item
This food item is a leftover	0.047	0.096*	-12.428	-16.115
from previous meals? (yes, = 1; no, = 0)	(0.028)	(0.052)	(13.265)	(13.280)
# of food items in this meal	-0.008**	-0.019**	0.388	6.222*
that are leftovers from a previous meal	(0.004)	(0.007)	(3.296)	(3.013)
# of food items in this meal	-0.030*	-0.056	--	-8.810
that are saved to become a leftover	(0.017)	(0.044)		(5.755)
# of food items selected	-0.002	-0.012**	-10.133**	-22.981***
for this meal	(0.003)	(0.005)	(4.204)	(5.466)
Hundreds of kcal selected	0.007*	0.013*		15.594***
for this meal	(0.004)	(0.006)		(3.752)
If this meal happens on a	0.006	0.002	-3.955	6.513
weekend (if yes = 1; if no = 0)	(0.014)	(0.039)	(7.458)	(8.236)
Item’s energy intensity	-0.008*	-0.018**	10.412***	10.248***
(Kcal/gram)	(0.004)	(0.007)	(1.431)	(1.634)
Relative humidity–daily min	-0.000	0.000	0.602***	0.608***
	(0.001)	(0.001)	(0.180)	(0.188)
Hundreds Kcal from all the	--	--	-2.478	= =
food items in this meal except for this particular item			(2.188)	
Food type (default = Dairy and Egg Products)				
Baked Products	0.013	0.050	59.806**	49.941**
	(0.018)	(0.044)	(22.965)	(18.363)
Cereals and pasta	0.021	0.023	112.709***	100.461***
	(0.024)	(0.036)	(22.564)	(21.283)
Fast Food & Restaurant	0.062**	0.092*	192.551***	117.023***
	(0.029)	(0.046)	(17.419)	(18.231)
Fruits and Fruit Juices	-0.002	0.004	-8.934	-7.706
	(0.015)	(0.042)	(18.312)	(18.605)
Nut Seed Legumes	0.037	0.037	21.933	26.432
	(0.027)	(0.049)	(22.920)	(23.766)
Oils Sauces Spices Herbs	0.044	0.114	-40.312***	-46.522***
	(0.026)	(0.068)	(8.626)	(6.971)
Poultry Sausages Pork Beef Fish Lamb	-0.009	-0.008	62.657***	60.956***
	(0.010)	(0.030)	(19.062)	(15.526)
Sweets	0.003	-0.002	26.013	14.097
	(0.014)	(0.035)	(21.226)	(14.582)
Vegetables and Vegetable Products	0.014	0.004	-59.057***	-46.821***
	(0.018)	(0.030)	(12.499)	(15.264)
Meal (default = Breakfast)				
Dinner	-0.003	0.016	43.775***	21.508**
	(0.016)	(0.036)	(13.567)	(8.656)
Lunch	0.026	0.074*	49.769***	21.360*
	(0.023)	(0.043)	(16.135)	(10.508)
Snack	0.018	0.039	4.422	14.028
	(0.018)	(0.039)	(13.191)	(11.488)
Constant	-0.014	-0.047	81.791***	74.860***
	(0.037)	(0.080)	(23.884)	(24.132)
Individual Fixed Effects	Yes	Yes	Yes	Yes
Study Day Fixed Effects	Yes	Yes	Yes	Yes
Observations	1,127	1,127	1,127	1,127
R-squared	0.216	0.231	0.407	0.469

OLS is Ordinary Least Squares. LPM is Linear Probability Model. Robust standard errors clustered at the subject level are in parentheses.

## Results

### Leftover selection

Table 1 classifies items selected for meals by the sample into six core groups according to whether the selected item was a leftover, whether selected items were fully consumed and, for items not fully consumed, whether they become leftovers. The modal selected item, representing 72.3% of items, was not a leftover and was fully consumed. There is a marginally significant difference in the disposition of selected items according to whether they were a leftover. For example, about 90% of leftovers that are selected are fully consumed, while 82% of selected items that are not leftovers are fully consumed.

Of the 1,177 selected solid food items, 12.15% of items and 9.71% of the calories were denoted as leftovers from previous meals (Table 2). Leftover selection varied significantly by food type–selected items that were vegetables and vegetable products (19.22% of items), breakfast cereals, grains and pasta (16.25%), meats and meat products (16.03%) were most likely to be a leftover. Leftover selection varied significantly by eating occasion with items selected for lunch (19.11% of items) most likely to be a leftover. Leftover selection was significantly different across the days of the week with the highest percentage of leftover items selected on Mondays (23.75% of items) and the lowest percentage on Sundays (6.15%). Men were more likely to report that selected items were leftovers (21.2% vs. 10.47% of items), though we note that only three of the 18 subjects were male. Leftover selection was significantly associated with age with older subjects reporting more leftover selection.

While the association between leftover selection and an item’s waste rate was not significant, we do notice a pattern in which the percent of leftovers are highest among those items with more than 75% of the selected material recorded as plate waste and those items with no recorded plate waste. Leftover selection was significantly associated with the meal’s total energy (calorie) content with leftovers most likely to be selected for meals featuring 500 to 749 calories.

### Saving leftovers

Of the 197 items that were selected but not fully consumed, 24.37% of items and 28.06% of the calories were saved to become leftovers (Table 2). The pattern of saving an unconsumed item to become a leftover featured fewer significant associations with the categories previously discussed. There is no significant association by food type, eating occasion, or gender. There is a significant pattern associated with age with the highest rates of saving leftovers among the oldest category (50%). Two significant patterns emerge with respect to day-of-the-week, with fewer unconsumed items being saved to become a leftover on Saturday, Sunday, and Monday, and with respect to calories selected for the eating occasion, with unconsumed items most likely to be saved for leftovers from eating occasions featuring more than 750 calories.

### Leftovers by eating occasion

Among the 307 meals recorded, 11.73% contained at least one selected item that was a leftover (Table 3). Among meals containing at least one selected item that was a leftover, the percent of the meal’s calories from leftover items was 79.19%. Among all meals, 5.87% ended with the creation of at least one leftover; among meals that featured at least one unconsumed item, 19.77% resulted in the creation of at least one leftover. Only one of the 41 snacks recorded (2.44%) featured a selected item that was a leftover.

### Regression analysis

Due to our inability to determine whether unconsumed items that respondents say they intend to save for later use are actually consumed, the regression analyses in Table 4 consider only items that were not saved for later use. Models A and B in Table 4 will be discussed together as they are related. Model A is an ordinary least squares regression model of the waste rate for each item selected by all respondents during the course of the study where waste rate is the fraction of the selected grams of the item that remain at the conclusion of the meal. This dependent variable is of interest for those concerned about the total mass of plate waste created. The dependent variable in Model B equals 1 if any of the selected item remained at the completion of the meal and equals zero otherwise. This dependent variable is of interest for those concerned with meal planning and consumption behavior that results in any mismatch between the amount selected and the amount consumed. Hence, for both Models A and B, positive coefficients suggest that there is a positive association between the amount of plate waste for this item at the conclusion of the meal and the independent variable, while negative coefficients suggest the opposite.

The first two rows of the table address variables that capture the association of leftovers with the waste of a particular item. The first row is an indicator variable that equals one if the item itself is a leftover and equals zero if the item is not a leftover. In both Models A and B the regression coefficient is positive, which is consistent with a greater rate of waste (Model A) and a higher probability that some of the item remains uneaten at the conclusion of the meal (Model B). The coefficient only reaches marginal statistical significance in Model B. The second row captures the total number of items on the plate for this meal that are leftovers, and in both Models A and B there is a significant negative relationship. Hence, holding constant whether the focal item was a leftover, if there were more total leftover items elsewhere on the plate, there was significantly less waste for the focal item.

The third row captures a variable that counts the total number of items that remain on the plate at the end of the meal that are being saved for later use, i.e., newly created leftovers. The negative, significant coefficients suggest that when items on the plate are being saved as a future leftover, the focal item features a lower rate of waste (Model A, marginally significant). Rows 4–6 in the table capture other relevant features of the meal, i.e., the number of items in the selected meal (row 4), the number of calories selected for the meal (row 5) and if the meal occurs on the weekend (row 6). An item has a lower probability of being wasted as the number of food items increases and a marginally higher probability of being wasted as the total number of calories selected increases.

Row 7 captures the association between the item’s energy density and waste; items with more calories per gram are associated with less waste (both Models A and B). Row 8 reveals no significant association between the humidity on the study day and subjects’ waste decisions.

Waste is defined as selection less consumption, hence we estimate models that provide insight into the functional building blocks of the waste outcome. Model C captures the relationship between these same independent variables and the amount of the food item selected for the meal (including any ‘seconds’ or additional servings), while Model D captures the relationship with the amount of the item consumed during the meal.

There is no significant association between an item being a leftover and the amount of that item that is selected or consumed (row 1) for models C and D. As more of the total number of items selected are leftovers, there is a marginally significant positive association with calories of any particular item that is consumed (Model D, recall most items are not leftovers), but not the calories selected (Model C).

The number of food items selected for the meal is negatively associated with the amount of this particular item selected and consumed while selecting more caloric meals is associated with consuming more calories of any particular item (Model D). Food items that are more energy dense are positively associated with the amount selected and consumed. The minimum humidity of the study day is positively associated with the amount of the item selected and consumed. Several significant relationships exist between the remaining variables and the amount consumed. The amount of an item selected and consumed is significantly higher than the omitted category of dairy and egg products if it was from a fast food or other restaurant or was from one of several food groups (pastas/cereals, baked products, and meats) and significantly lower if it was from two other categories (oils/sauces/spices/herbs or vegetables). Compared to breakfast, dinners and lunches are associated with significantly more calories selected and consumed.

## Discussion and conclusions

We identify several significant patterns concerning leftovers among the 18 participants who tracked their daily food selection, intake and plate waste for about one week. Importantly, if these findings generalize to broader and more representative populations, than many of these patterns are actionable and offer insight on who to target to reduce food waste and when to deliver intervention strategies. First, there is evidence that selecting leftovers to be part of a meal affects the plate waste associated with that meal. Holding all else equal, there is a marginally significant 9.6 percentage point increase in the probability that a selected item is not fully consumed if it is a leftover compared to an item that is not a leftover (Table 4, Model B). However, the evidence about item plate waste is mixed. The average percent of the item that was wasted (Table 4, Model A) was not significantly different from items that were not leftovers. To envision this pattern, imagine 10 plates of chicken. If the chicken selected for the meal was leftover, imagine 5 clean plates and 5 plates with two ounces of chicken remaining at the end of the meal. If it was chicken prepared for the first time, imagine you would have 9 clean plates and 1 plate with 10 ounces chicken as plate waste. Hence, both the leftover and freshly prepared chicken feature the same average amount of chicken as plate waste (one ounce), but the frequency of plate waste would be five times higher for the leftover chicken.

Leftovers also have effects that spillover to other items selected for a meal. When the other items selected for a meal feature more leftovers, the focal item is less likely to generate plate waste and a larger amount of the focal item is consumed. For example, suppose freshly prepared peas were selected for a meal. The peas would be consumed more and result in less plate waste if they were surrounded by a leftover pork chop and leftover potatoes than if they were surrounded by a freshly prepared pork chop and a freshly prepared potato. This would be consistent with subjects assessing the relative freshness of items on the plate and increasing their consumption focus on an item if it is surrounded by leftovers. It also offers strategies to reduce food waste among items that are served as leftovers. If the data are causal and the results generalize to other populations, then saving and preparing meals that are comprised entirely of leftovers will result in less food waste. Such a pattern was common among the sample considered for this study: for meals that included at least one leftover, about 80% of the calories selected were leftovers.

As subjects identify more items on a plate that they intend to save for a later meal, they generate marginally less waste for this particular item. Consider an example where a subject has a plate with lasagna, garlic bread, and a green salad. The subject wastes less of the salad if they have marked the lasagna as an item they want to become a leftover, while the subject wastes even less of the salad if both the lasagna and garlic bread were to be designated as a future leftover.

Nonetheless, since not all of the leftovers will be consumed, and are less likely to be fully consumed than non-leftover items, a more effective strategy for reducing food waste may be to train people to prepare and select less food (portion and meal size reduction). Indeed, Models A and B in Table 4 confirm that the amount and frequency of plate waste significantly increase as meal size (as measured by calorie content) increases.

Alternatively, strategies that improve the appeal of leftover items (e.g., storage tips and creative recipes that use leftover items) could also help reduce the amount of food wasted. However, if campaigns are successful at reducing meal sizes, fewer leftovers will be created, rendering campaigns to improve the appeal of leftovers less efficacious simply because fewer leftovers will be created. That is, a clever recipe to use leftover chicken may be forgotten if the strategy to limit portion sizes reduces the frequency of leftover chicken from, e.g., a weekly to a monthly occurrence.

Leftover vegetables, meats, and cheeses were frequently selected and at least two of these food groups have large environmental [29] and financial impacts [30]. Due to not all leftovers being saved, targeting the preparation and selection of smaller portions of these foods and smaller meals in general could results in cost and environmental savings.

Younger subjects (<40 years) less frequently selected and created leftovers, demonstrating that targeting these age groups could result in a larger effect size when interventions focused on saving and consuming leftovers are tested to reduce food waste. Significant associations with the eating time, day, and occasion also suggests possible ideas for leftover utilization. Among our sample, Monday and Friday lunch were the most popular meals for selecting leftovers, with more than one-third of all such meals containing at least one leftover item. Other weekday meals and weekend eating occasions contained significantly fewer leftover items, suggesting that strategies that can help consumers leverage leftover items outside of the lunch meal may provide another avenue for increasing the utilization of leftover items.

Furthermore, unconsumed restaurant and fast food items featured among the lowest rate of being saved for leftovers (10.3% vs. 24.4% overall). This is consistent with insights from the literature [31] and suggests that strategies that promote and facilitate leftover saving and consumption from food service settings may pay dividends.

While the data and analyses provide a first glimpse at more nuanced view of leftover selection and creation, we must recognize several limitations of the study. First, this study featured only 18 predominantly female participants from a single geographical location in the United States. Second, given the small sample size, we do not have enough power to derive robust conclusions regarding the food items saved for later use. Also, the current methods are unable to determine the percent of all stored leftovers that are selected for meals. For example, while we find that 24% of all unconsumed items are saved to become a leftover, we do not know if all of these items were eventually served–some might have been saved and then discarded after the study period concluded without ever being selected for a subsequent meal. Likewise, the methods do not include an inventory of potential leftovers at the beginning of the study period that would permit calculating the percent of leftover items are eventually selected for a meal during the study period.

## Supporting information

S1 Data (CSV)
